# The Impact of Digital Transformation on Inpatient Care: Mixed Methods Study

**DOI:** 10.2196/40622

**Published:** 2023-04-21

**Authors:** Philipp Koebe, Sabine Bohnet-Joschko

**Affiliations:** 1 Faculty of Management, Economics and Society Witten/Herdecke University Witten Germany

**Keywords:** digital transformation, digitization, health care provision, hospital, trends

## Abstract

**Background:**

In the context of the digital transformation of all areas of society, health care providers are also under pressure to change. New technologies and a change in patients’ self-perception and health awareness require rethinking the provision of health care services. New technologies and the extensive use of data can change provision processes, optimize them, or replace them with new services. The inpatient sector, which accounts for a particularly large share of health care spending, plays a major role in this regard.

**Objective:**

This study examined the influences of current trends in digitization on inpatient service delivery.

**Methods:**

We conducted a scoping review. This was applied to identify the international trends in digital transformation as they relate to hospitals. Future trends were considered from different perspectives. Using the defined inclusion criteria, international peer-reviewed articles published between 2016 and 2021 were selected. The extracted core trends were then contextualized for the German hospital sector with 12 experts.

**Results:**

We included 44 articles in the literature analysis. From these, 8 core trends could be deduced. A heuristic impact model of the trends was derived from the data obtained and the experts’ assessments. This model provides a development corridor for the interaction of the trends with regard to technological intensity and supply quality. Trend accelerators and barriers were identified.

**Conclusions:**

The impact analysis showed the dependencies of a successful digital transformation in the hospital sector. Although data interoperability is of particular importance for technological intensity, the changed self-image of patients was shown to be decisive with regard to the quality of care. We show that hospitals must find their role in new digitally driven ecosystems, adapt their business models to customer expectations, and use up-to-date information and communications technologies.

## Introduction

### Background

Digital transformation is permeating all areas of society [1,2]. Scientific circles also refer to it as the Fourth Industrial Revolution [3]. On the basis of the latest advancements in medicine and in the context of general technological progress, digital trends also influence the provision of services in health care [4-7]. Health systems have become the focus of political and social debates in industrialized Western countries for a number of reasons [8,9]. Demographic developments have led to an increase in life expectancy [10]. This increase in life expectancy is primarily due to the medical-technical progress in the past as well as the comprehensive access to health care services in most high-income Western countries [11]. The result is an expansion in the use of services with an upward trend [12]. Furthermore, high life expectancy and the associated decline in the birth rate have led to a shift in the overall population structure in recent decades [10,11]. Fewer younger people have to care for a growing proportion of older people. This is already resulting in numerous bottlenecks [13]. Certain constraints include the financing of increasing health and nursing care services, the provision of comprehensive care even in less populated regions, and the staffing of health and nursing care facilities [14,15]. Owing to the personnel intensity of health services, many industrialized Western countries are already dependent on immigration for medical professionals. The use of new technologies and digital applications can reduce the financial and personnel burden on health care systems, especially on inpatient care [4-6,16].

On the one hand, there are numerous current trends resulting from digital transformation [17]. Artificial intelligence (AI), data analytics, further automation and robotization, and cloud computing or the Internet of Things (IoT) are driving digital permeation in society and the economy [17-20]. This is also accompanied by visionary technologies such as digital twins for use in virtual worlds such as the metaverse [18,21]. These trends are also creating a greater need for cybersecurity.

**Figure 1:**
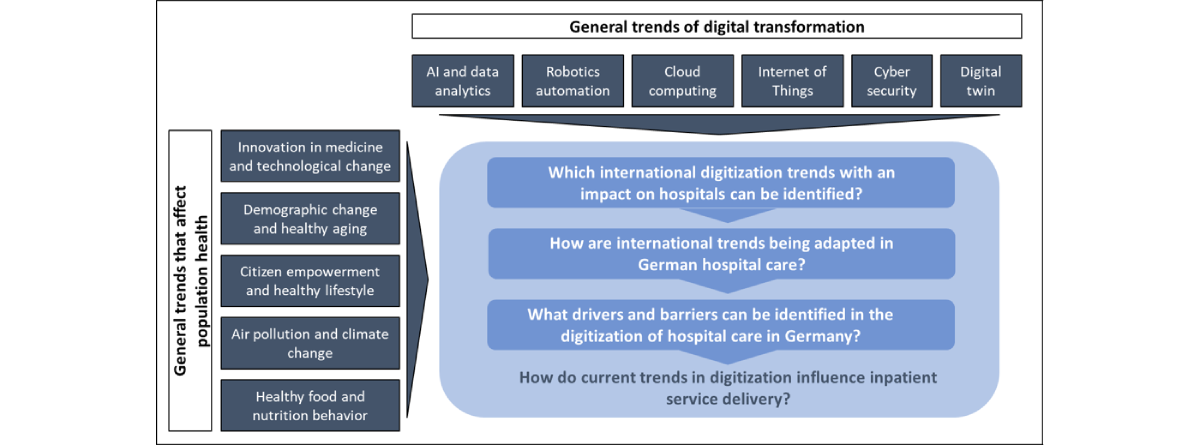
Derivation of the research objective. AI: artificial intelligence.

In contrast, general trends related to population health are leading to changing framework conditions [12,13]. Leap innovations in medicine and technological change, the aforementioned aging population with a need for healthy aging, the empowerment of the population, changing lifestyles, and healthy nutrition are influencing current developments in health care [22-24]. Environmental influences such as air pollution and climate change are also creating pressure for action in some health care fields [25-27]. As a result of these trends, various questions are being asked to better assess and control future developments. We make a special reference to German health care delivery as the German hospital and health care system can definitely be seen as a reference model for many other European and international health care settings [28,29]. This study considered the emerging trends for the future provision of care in hospitals and beyond in a patient-centered or outcome-oriented health care system that incorporates digital technologies [30-32]. Figure 1 shows the relationships between the general trends that have developed, with the purpose of deriving the research question.

### Objectives

Our central research question addressed how current trends in digitization influence inpatient service delivery. We approached this question in 3 steps. First, we identified the international trends in digitization that have an impact on the hospital sector. Second, we addressed the adaptation of these trends with regard to the German hospital sector. Finally, we derived drivers and barriers and concluded which developments result from these trends.

## Methods

### Overview

**Figure 2:**
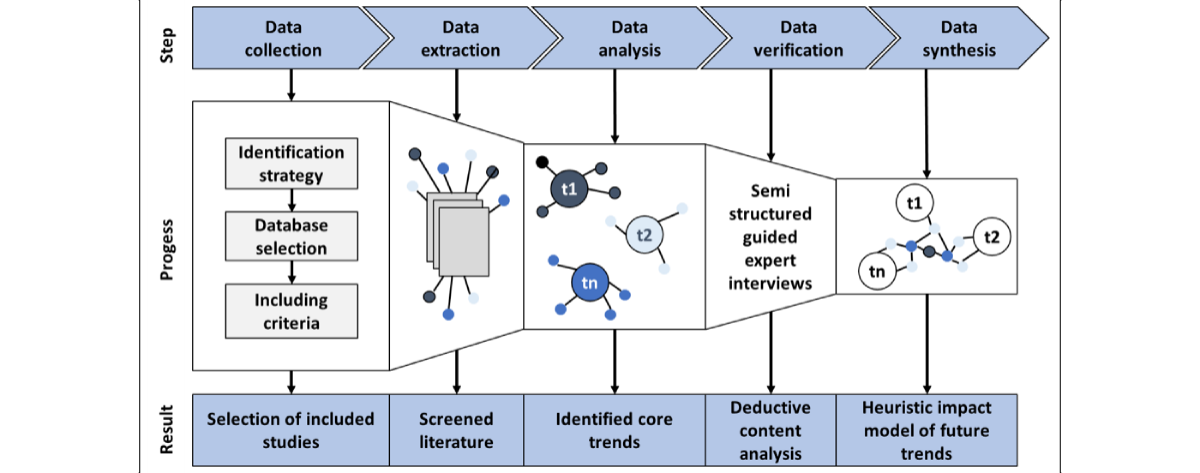
Research methodology.

We conducted a sequential study. Our mixed methods approach consisted of a scoping review and a qualitative survey. Our research design was based on 5 steps, as shown in Figure 2. The first step involved fundamental data collection. This resulted in the inclusion of studies, which was the starting point of our analysis. In the second step, data extraction took place. The available studies were compared, suitable classification features were determined, and their contents were exploited. The third step was data analysis and synthesis using a narrative approach, resulting in core trends. In the fourth step, a classification, verification, and discussion of the selected trends took place. The data obtained were subjected to a deductive content analysis. In the fifth step, a higher-level synthesis of both data collection points was performed. A heuristic logic model was used as an instrument.

### Scoping Review

#### Search Strategy

**Figure 3:**
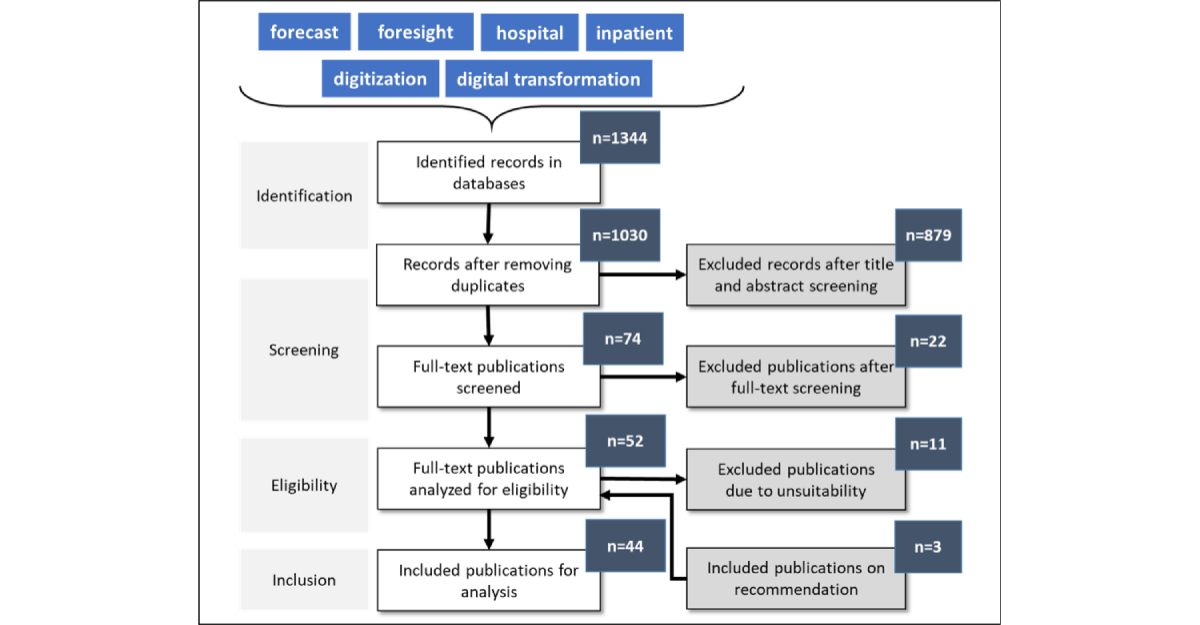
Flowchart of literature search strategy.

The scoping review is reported according to the PRISMA (Preferred Reporting Items for Systematic Reviews and Meta-Analyses) guidelines [33]. Accordingly, the following work stages were fulfilled: (1) definition of the research question, (2) systematic search, (3) selection of relevant studies, (4) data extraction, (5) data analysis and synthesis, and (6) interpretation of the results. We conducted our scoping review based on the extended current recommendations [34,35] and presented the results of our search strategy in a flowchart (Figure 3). Between October 2020 and February 2021, we conducted a systematic search of the following databases: PubMed, MEDLINE, EconLit, EconBiz, and ScienceDirect. An initial search was conducted in September 2020 to select an appropriate search strategy. Our search was limited in time to the last 5 years. Papers published between 2016 and 2021 were included. The inclusion of earlier papers would lead to bias in the results as the consideration of trends is only meaningful over a manageable period in the past [36,37]. The language was restricted to English. After screening the titles and abstracts, all articles dealing with future trends as well as those dealing with inpatient care or the service provision of the health care system and digitalization were included in the full-text search. In total, 2 reviewers performed the full-text screening according to these inclusion and exclusion criteria. Disagreements were discussed between the 2 reviewers to reach a consensus. In the case of a possible disagreement, a third author was appointed.

Our scoping review referred to current and future trends related to digital transformation on the one hand and service delivery in hospitals on the other. The words “forecast” and “foresight” were searched in combination with other terms (eg, “digital transformation,” “digitization,” and “hospital”). The search terms used in the various databases are shown in Multimedia Appendix 1. In a further step, the reference lists were searched backward for other relevant publications not listed in the aforementioned databases. In our review, we also included manuscripts that were recommended by authors or experts. Some search terms were discarded in the process of developing a search strategy. For example, the search terms “trend” or “future” were not included as they were too nonspecific and triggered too many irrelevant hits. The concept of service provision also had to be differentiated more precisely. The search strategy was ultimately refined to focus on hospitals.

#### Inclusion Criteria

Articles were included if the following 3 factors applied: a reference was made to the future or trends, a reference was made to the terms “digital” or “technology,” and a reference was made to inpatient service provision or hospitals. The documentation is shown in Multimedia Appendix 2 [20,38-80]. Articles were excluded if the terms “trend” or “future” were used to refer to a possible new treatment or diagnostic procedure; the prediction of the spread of disease; or specific developments in the fields of brain, genetic, or biotechnology research. Very frequently, the overall search showed the use of AI and machine learning (117 studies); IoT, wearables, and mobile health apps (86 studies); or big data (70 studies). Publications from a medical discipline or on medical indications were not included. The most common indications were related to cardiovascular disease (79 studies); oncology (96 studies); or imaging, particularly radiology (89 studies). However, of these, 1 study was included that was related to the organization of radiology departments in hospitals. Psychiatric indications and mental health were also not included in the study. Case studies and feasibility studies related to individual countries or organizations were also excluded, as were studies on trends in education and training in clinical settings (eg, the future of medical staff training). Publications related to the COVID-19 pandemic were not included. In total, the search provided 77 studies linked to COVID-19. The particular use of technology, such as blockchain or cybersecurity, was only included when there was genuinely a direct link to inpatient care. This occurred in only 1 case.

#### Study Inclusion

The identified articles from all databases were uploaded to the EndNote software (Clarivate Analytics), and duplicates were removed. In total, 2 researchers independently performed the first step of the screening process based on titles and abstracts, as reported in Multimedia Appendix 3. In the second step, 2 independent researchers carefully read the articles with available full texts to decide which would ultimately be included in the systematic review. Articles that met the eligibility criteria were selected for inclusion in the systematic review. The reasons for the exclusion of full-text articles were recorded and included in the PRISMA flow diagram [33]. The reference lists of the included articles were carefully searched by hand to identify other appropriate articles. In total, 2 researchers independently extracted the following data from each article: (1) study identification (study author and year of publication), (2) main trend of the study (technology, innovation, or process), and (3) key statements of the study (fields of application, opportunities, risks, and recommendations for action).

Any discrepancies that arose during the data extraction process were clarified through discussion among the authors. Subsequently, the extracted contextual factors were labeled according to their topic, and the studies were summed up so as to show the respective factors. In addition, the studies were classified using 2D scales. The first dimension described the inclusion of the user within the trend. The second dimension related to the intersectoral component. Furthermore, the trend factors were grouped into the 3 application fields: planning and steering, core clinical processes, and communication and data connection. For each of these areas, individual trend factors were described as having an impact on inpatient care. Each factor was double-checked by the 2 researchers, and incongruities were resolved through discussion. We used a narrative approach to synthesize the data [81]. Finally, the results of the synthesis were presented in a trend chart to contextualize the trends’ influences on other trends and illustrate the factors affecting each trend. In addition, barriers that contributed to the deceleration of the trends were extracted from the studies.

### Expert Interviews

The identified trends were further processed for in-depth analysis and verification. The transfer of the trends that were extracted based on international scientific literature resulted in a qualitative survey of experts [82]. In this methodological component of our study, we focused on the German setting. Later, we will put these results back into a generalizable context so that the findings can also be applied to a broad spectrum of other countries. The expert interviews were conducted in the form of semistructured, guide-based interviews [83]. The interview guide was largely based on the identified core trends. The experts were selected based on the 2 decisive criteria of digitization experience in the health care sector and experience in or knowledge of the area of service provision.

Experts from 4 specific groups were interviewed in equal numbers: first, the group of inpatient care suppliers; second, the group of digital service providers in the health care industry; third, overarching institutions in the health care sector; and fourth, the group of relevant scientists. The number of interviews that needed to be conducted was not determined in advance. After 12 interviews, information saturation was reached [84]. The guide was tested for suitability in advance in a pretest with 3 participants and adjusted before final use. The data generated over the course of the interviews were analyzed deductively [85,86] in a sense-reconstructive, interpretative manner. The statements to be analyzed were compiled using the MAXQDA software (VERBI GmbH). The data analysis was performed by 2 independent researchers following a theory-driven definition of structuring dimensions to form the main categories and subcategories. An iterative process was used to establish definitions, identify anchor examples, and generate coding rules. The generated codes were subjected to a reliability test in several steps until the summary test of all the quality criteria was completed.

### Data Synthesis

The extracted data from the literature and the obtained research data from the interviews were synthesized into a heuristic logic model [87,88]. Heuristic logic models are used in designing systematic review questions and collecting research results and as analytical tools [89]. Analytical logic models demonstrate a logical chain between inputs and outcomes. When the influences of the overarching relationship are demonstrated, it may not be necessary to examine all the individual components and their connections. We show a logical sequence of effects, including possible adverse effects, that influence inpatient care in a chain of identified trend characteristics. The model was used to illustrate the authoritative impact of the core trends on inpatient service delivery. This enabled the sketching of a trend development corridor and a more detailed description of the impact relationships [90]. Finally, the decelerating factors were also shown based on the data material.

### Ethical Considerations, Informed Consent, and Participation

The study did not meet the need for ethical review as it did not involve special categories of personal data. All procedures performed in this study were in accordance with the ethical conducts of the General Data Protection Regulation and *Bundesdatenschutzgesetz* (German implementation of European Union regulations). Email invitations were sent to prospective candidates. The invitation included an overview of the project and the research scope. The voluntary nature and anonymity of participation were pointed out, together with complete information on the European Union General Data Protection Regulation. We requested written informed consent from all participants to record the interviews and use the information provided in our research.

## Results

### Results of the Scoping Review

The search strategy identified 1344 articles from the 5 databases, as shown in detail in Figure 3. After duplicates were removed, 76.64% (1030/1344) of the articles were retained for title and abstract screening. A total of 74 articulated full-text articles were read. After screening, 59% (44/74) of the studies [20,38-80] met the eligibility criteria for this systematic review.

The main characteristics of the included studies are listed in Multimedia Appendix 4 [20,38-80]. The classification into the topic field was performed to categorize a rough content assignment of the article. The core statements reflected the main contribution of the article and were later used to categorize the trends. The topic of AI and machine learning was addressed in 14% (6/44) of the studies [47,49,57,58,61,69] in different contexts. A total of 30% (13/44) of the studies [42,43,48,51,52,60,64,66,67,72,76,79,80] were originally related to data-driven topics (eg, data-driven resource use). The change in processes used by digitization was the subject of 25% (11/44) of the studies [40,44,46,50,55,63,68,70,74,75,78]. Among other things, processes in inpatient care that use electronic health records played a role. Mobile apps and IoT were the subject of 14% (6/44) of the studies [20,39,41,59,62,71]. These studies also showed close connections to data use and process redesign. The use of robots in clinical settings was addressed in 7% (3/44) of the studies [38,45,54]. The establishment of platforms or the development of a digitally connected ecosystem was considered more closely in 9% (4/44) of the studies [53,65,73,77]. A total of 2% (1/44) of the studies dealt explicitly with personalized medicine [56]. However, this topic was also addressed in numerous other studies (eg, with the use of AI or IoT). In all the studies, trends were described, and future developments were derived from them. In addition, they identified problem areas that will be compared at a later stage in this paper as internal and external trend barriers. In some cases, recommendations for action were derived or inferred from the barriers.

From the included studies, 8 trends were identified that affect hospitals as a result of digital transformations. For this purpose, 44 studies [20,38-80] were analyzed, the core topics were classified, the most important technologies were pinpointed, and the fields of application and hurdles were extracted, as reported in Multimedia Appendix 5 [20,38-80] (Textbox 1).

The core trends are presented in Table 1. In addition to naming the trends, the essence of each trend is described, as well as the most important and relevant technologies and the affected application fields within the hospital setting.

**Figure 4:**
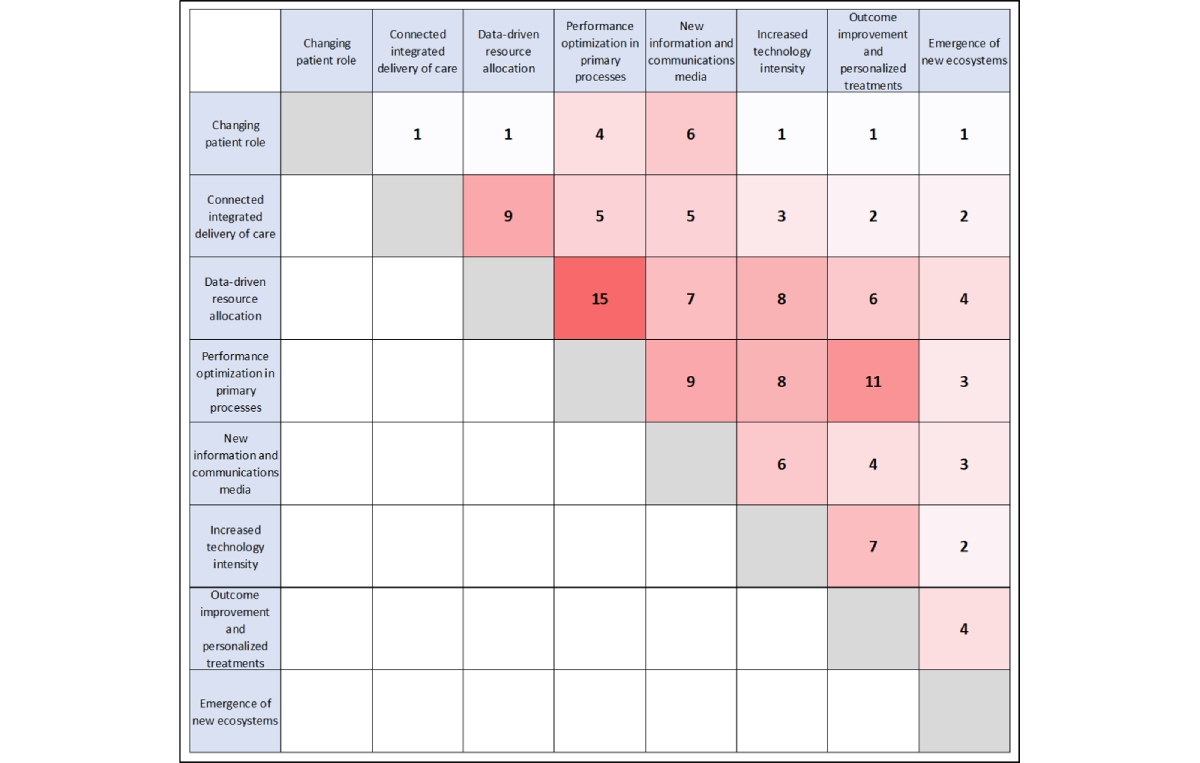
Connections between the trends.

To illustrate the interrelationships between the individual trends, we presented the connecting factors in a matrix according to the number of times they were mentioned in the studies. Figure 4 illustrates which trends have a close linkage and shows the intensity with increasing red coloring. The closest links are between trends 3 and 4, with 15 common factors. Dense relationships were also evident between trends 4 and 7. Trend 1, in contrast, shows the fewest connections with other trends. The matrix represents an initial basis for further processing in the synthesis of the results, which will later also include the results of the interviews.

**Trends identified from the systematic review.**

**Trend 1—changing the patient role**

- Patients are taking on a new role in the entire care process from prevention to diagnosis and therapy. They participate in decision-making, generate and use data, and have transparent information on diseases and available health care services. The most important technologies are the internet, smart devices, and mobile health apps.

**Trend 2—connected, integrated delivery of care**

- Connected technologies and cross-provider data exchanges promote intersectoral collaboration. Providers exchange and share data across the care process. Important technologies include electronic health records, the Internet of Things (IoT), and e-prescriptions.

**Trend 3—data-driven resource allocation**

- A data-based allocation of resources by means of predictive models enables the precise planning and control of care capacities. As a result, available resources are better allocated and used across an organization. Consequently, waiting times can be reduced, and patient throughput can be optimized. The most important technologies are data mining, machine learning, and predictive analytics.

**Trend 4—performance optimization in primary processes**

- The performance of core clinical processes in diagnostics and therapy is optimized. This involves using available data in real time to deploy equipment, rooms, or personnel efficiently. Relieving systems are used for the staff. In addition, services are patient-centric to meet the individual needs of patients within an institution. The most important technologies are electronic health records, machine learning, and assistive robots.

**Trend 5—new information and communications media**

- Emerging information and communications technologies are transforming the interactions between all stakeholders in the care process. They promote information transfer and facilitate the understanding of complex interventions through visual media. The most important technologies are avatars, mobile health, and IoT.

**Trend 6—increased technological intensity**

- The intensity of technology is increasing rapidly at all levels. The hospital is becoming a high-technology digital institution. Intensive data use leads to intensified collaboration with digital service providers and the extensive use of software or algorithms. The most important technologies are smart devices, robots, and cloud services.

**Trend 7—outcome improvement and personalized treatments**

- The quality of outcomes is rising in the context of increasing data use and the associated optimization of treatment procedures focusing on predictive forecasting models for the early detection of diseases. The perspective of personalized diagnostics and therapy is at the heart of the treatment process. The most important technologies are data analytics, machine learning, and predictive medicine.

**Trend 8—emergence of new ecosystems**

- Digital networking is leading to new providers and new business areas so that health care is evolving into an ecosystem in which established service providers have to maintain their competitive position and find their role in providing care. Platforms for exchanging data and mediating health care services play an important role in these ecosystems. The most important technologies are mobile health, machine learning, and predictive analytics.

**Table 1:** Identified trends in the scoping review.

Trend number	Core trend	Description	Key technologies	Fields of application
1	Changing the patient role	Patients have an active role in the entire care process. They participate in decision-making processes and have comprehensive transparency in their own data and available services.	Internet, smart devices, and mobile health	Decision support [78] Increase in patient compliance [63,71] Integration into the therapy and monitoring process [68]
2	Connected and integrated delivery of care	Service providers collaborate without sector barriers. They are connected to each other and provide all necessary diagnostic and therapeutic information to one another.	EHRs^a^, smart devices, IoT^b^, and e-prescriptions	Integrated data exchange between providers [77] Full use of the EHR [46] Transmission of upstream and downstream treatment information [51,66]
3	Data-driven resource allocation	The capacities of service providers are optimally used. They analyze real-time data and make decisions based on currently available resources beyond their own institution.	Big data, machine learning, data mining, EHR, HISs^c^, and predictive analytics	Process optimization [42,48] Planning and control of resources by data-driven decision models [44,48,52,55] Predictive forecasting for capacity planning and the identification of bottlenecks and potential risks [48,52,69,80]
4	Performance optimization in primary processes	Hospitals manage their internal processes efficiently and in a patient-centric manner. They use technology to support staff in diagnostics and therapy decisions.	EHRs, robotics, IoT, machine learning, and data platforms	Development of personalized treatment pathways [42,44] Optimization of diagnostics and outcomes across the care continuum [50,58,76,79] Robotics in several clinical areas [38,45,54]
5	New information and communications media	Service providers are broadening the range of information and communications channels. They create shared and low-threshold ways to convey information and meet patients’ communication needs.	Avatars, virtual reality, augmented reality, EHRs, mobile health, and IoT	Transfer media for data exchange between providers and patients [41,77] Enhancing patient engagement [68,78] Creation of new barrier-free communication channels [63]
6	Increased technological intensity	Hospitals are deploying an increasing number of technological solutions. They are exploiting the potential of innovative technical applications and raising the level of technological complexity.	Smart devices, IoT, robotics, machine learning, and cloud services	Expansion of the inventory of hardware and technical equipment [38,54] Building partnerships with digital service providers [39,77] Connection to external systems for permanent data exchange in real time [48,57,77]
7	Outcome improvement and personalized treatments	The outcomes of patient care are steadily improving. Early detection and disease prediction systems enable an optimal treatment sequence according to the personalized needs of the patient.	Big data, machine learning, predictive analytics, personalized medicine, and 3D printing	Evaluation of incoming monitoring data from patients [20,50,62] Connection to databases and data platforms for the training of AI^d^ [58,61,79] Individual therapies [53,65,76]
8	Emergence of new ecosystems	Service providers are linked together in an ecosystem to provide care. They use platforms to exchange data and optimally allocate services to the right place at the right time.	Digital platforms, mobile health, machine learning, and predictive analytics	Appointment management [66] Reduction in waiting times [46,79] Assignment of appropriate experts [65,69] Distribution of available materials [69,73] Switching on of suitable gatekeepers [56,70]

^a^EHR: electronic health record.

^b^IoT: Internet of Things.

^c^HIS: health information system.

^d^AI: artificial intelligence.

### Results of the Expert Interviews

The qualitative approach to analyzing the impacts of trends brought to light additional aspects aside from the findings from the literature. On the basis of the core trends shown, Table 2 presents the positive and negative trend effects, conditions for trend realization, and trend drivers. This made it possible to describe additional characteristics for each individual trend, enabling a holistic approach to the impact mechanisms of the trends resulting from the digitization of inpatient care.

The changed patient role leads to an increase in transparency, more patient autonomy, better opportunities for participation in the treatment process, and an expansion of self-competence. However, in the case of an overload of information, this can also lead to excessive demands or the communication of incorrect information. Therefore, the decisive conditions are to strengthen trust in acting institutions and make information available through public authorities. The driving factors are good health literacy and a high level of digital competence. Connected and integrated cooperation between providers leads to better access to health care services and faster data exchange between them. However, this may lead to increased competitive pressure, resulting in the exit of market players. The key conditions for this trend are incentives to cooperate so that individual providers are not disadvantaged. To achieve this, compensation systems must be adapted so that network-integrated collaboration is worthwhile and the necessary interfaces for data exchange are available. Driving factors include easy market access and existing supply gaps. Data-driven resource allocation leads to increased efficiency, a better use of capacity, and offsetting of skill shortages. However, there are strong dependencies among service providers regarding the extent to which they are willing to collaborate and share information. The intensive exchange of data at various levels between institutions can lead to data protection and data security risks. The governing conditions include the availability of data, interfaces, and technical expertise. The driving factors include scarce capacities, high data density, and public data platforms.

The performance optimization of core clinical processes leads to higher productivity, increases in quality, and higher patient and employee satisfaction. In contrast, there is a high internal dependency on a wide range of factors and the need for a continuous process of adaptation and optimization. The decisive conditions are investments in new systems and the associated qualifications of the workforce. Acceptance and openness are essential for success. The driving factors are medical progress, falling costs of innovations, and new job profiles. New information and communications technologies are accompanied by a change in patients’ need to interact with providers. They lower communication barriers and lead to better information delivery. For clinics, the wide range of media may result in higher costs for their operation, and additional qualifications may be necessary. The key conditions are an understanding of communication needs and an acceptance of media use by all stakeholders. The driving factors are the digital competencies of all stakeholders and the current general societal trends in media preferences. Increasing technology intensity ensures higher productivity and compensates for a lack of skilled workers. This is accompanied by greater complexity and the associated requirements for cybersecurity and employee qualifications. Comprehensive long-term investments and the acquisition of new knowledge are required as decisive conditions. The driving factors are technological progress and rising patient expectations. The application of personalized medicine leads to an improvement in outcomes as well as the possibility of specialization and the opening up of new fields of treatment. In contrast, there is an adjustment of care-oriented business models, which, among other things, also include prevention to a greater extent. The key conditions are high data quality and availability, the market maturity of innovative technologies, and confidence in new treatment procedures. The driving factors are medical and technical progress as well as the service offerings of competitors.

**Figure 5:**
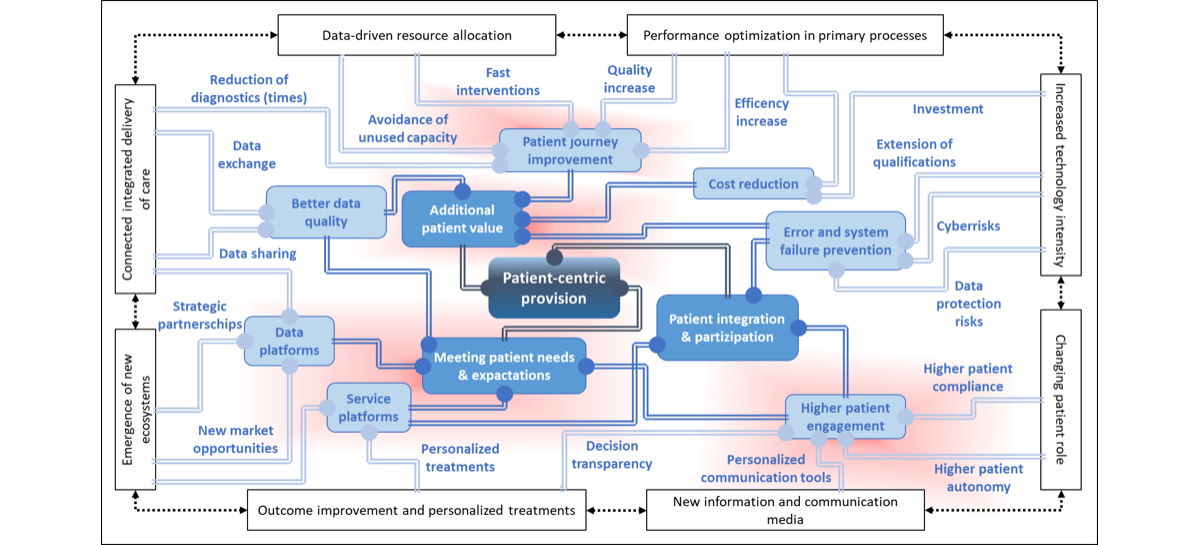
Heuristic impact model of digital transformation trends in hospitals.

The emergence of new ecosystems has led to new customer-centric health care services, an exploitation of the potential of the platform economy, and the associated opportunities to increase customer value. This creates new competitive situations that can put pressure on providers and lead to inappropriate power relationships among individual market participants. The decisive conditions are an understanding of digital ecosystems also on the part of the legislator, transparency, and fair conditions on platforms for all service providers. The driving factors are low barriers to market entry, little regulation, and a strong focus on customer needs. Figure 5 shows the trend correlations in a heuristic impact model.

**Table 2:** Trend characteristics from expert interviews in Germany.

Trend number	Core trend	Positive effects	Negative effects	Prerequisites	Trend drivers
1	Changing the patient role	Increasing transparency Growing patient autonomy Better participation opportunities Growth of self-competence	Risk of disinformation Overwhelming media diversity	Trust in institutions Public provision of information Comprehensive education	Health literacy Digital competencies Information access
2	Connected and integrated delivery of care	Better access to services Faster data exchange Reduction in interface problems	Additional competitive pressure between providers	Performance incentives Adjustment of reimbursement systems Data exchange interfaces	Easy market access Existing supply gaps Integrated planning tools
3	Data-driven resource allocation	Increasing efficiency Optimal use of resources Compensation for the shortages of skilled workers	Dependence on service providers Data protection risks Increasing complexity	Data availability Interface availability Specialist availability	Scarce resources High data density Public data platforms
4	Performance optimization in primary processes	Efficient provisioning Increasing quality Higher satisfaction (patients and staff)	Dependencies of many factors Constant change process	Investments in new systems Acceptance of new processes Permanent qualification	Medical progress Decreasing costs New professions
5	New information and communications media	Meeting the needs of patients Reduction in communication barriers Improving the availability of information Higher satisfaction	High expenditure for the maintenance and provision of the media Additional need for qualification	Acceptance of the parties involved Knowing and understanding needs Quality assurance	Digital skills (patients and staff) Demand for contemporary media from everyday social life
6	Increased technological intensity	Achieving advances in productivity Compensation for the lack of skilled workers Relief of the workforce	High effort for cybersecurity Increasing complexity Need for technical knowledge	Extensive investments Comprehensive qualification Acquisition of new areas of knowledge	Technical progress Patient expectations
7	Outcome improvement and personalized treatments	Increasing outcomes Development of new service areas Opportunities for specialization	Adjustment of the business model	Data quality and availability Market maturity of technologies Confidence in new treatment procedures	Medical progress Technical progress Competitors’ offers
8	Emergence of new ecosystems	Creation of customer-centric proposals Exploiting the potential of the platform economy Increasing customer value	Highly competitive pressure Inappropriate market power of some players Inadequate regulation	Understanding digitally controlled ecosystems Transparency of the service offering Fair conditions on platforms	Low barriers to market entry Minimal regulation High customer interest and demand

### Results of the Data Synthesis

The interrelationships between the trends are very complex and require an adequate form of representation to be able to understand the trend effects. We show these relationships in a heuristic impact model in Figure 5. The 8 core trends are plotted on the outer level. They form the starting point of the interaction. At the next level inward, the first-degree effects are derived. The links there are underpinned with the help of the extracted factors from the literature and the expert interviews. At the next level, in medium blue, the second-degree effects are shown. The characteristics of additional patient value, meeting patient needs and expectations, and patient integration and participation emerge. At the last level, the third-degree effect is derived, which provides patient-centric care. This representation illustrates the positive foresight of future development predicted in the literature and in the expert interviews. The data synthesis shows that, as a result of the digital transformation, the value of care for patients will increase considerably. The nodes highlighted in red show a concentration of related parameters. They are based on the trend correlations shown in Figure 4 and the qualitative responses from the interviews.

**Figure 6:**
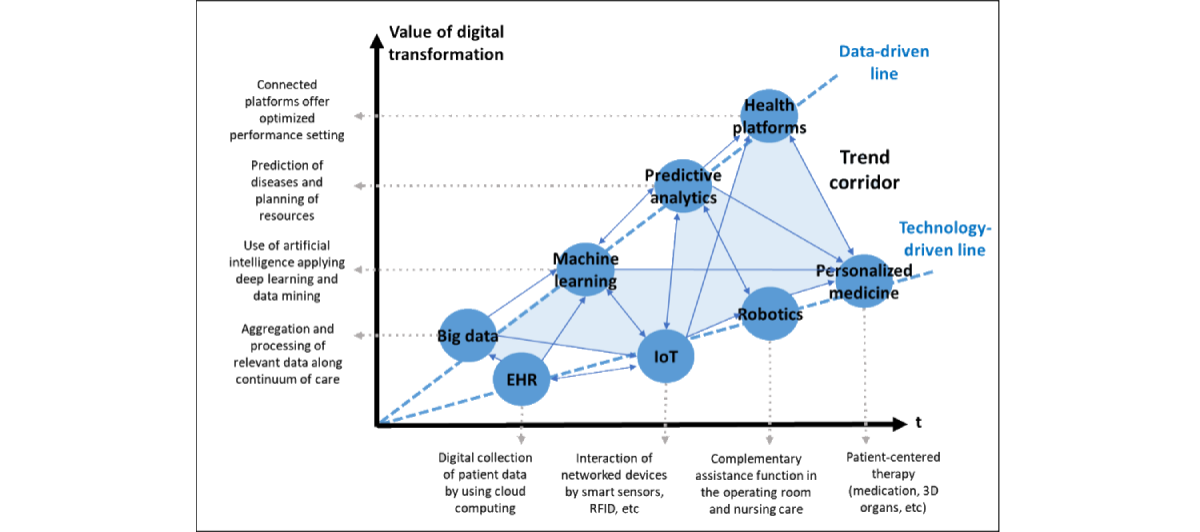
Schematic trend development perspective. EHR: electronic health record; IoT: Internet of Things; RFID: radio frequency identification.

For a more intuitive illustration of the trend development perspective, we placed the value of care as a result of digital transformation in a temporal context. Figure 6 shows the development horizon with increasing digital transformation progress and the associated added value. On the one hand, this is driven by technological intensification [38,39,41,45,54,59,71]. The primary manifestation of this is the use of digitally controlled and networked devices as well as software and algorithms. In contrast, a rise in the quality of care is to be expected [20,43,44,67,72,76]. The availability of large amounts of data enables better predictability of diseases and possible therapies and better control of the care itself. Machine learning leads to a continuous improvement in outcomes and decision-making [47,57,58,61,69].

The convergence of personalized medicine as a result of better diagnostics and individualized therapy decisions, incorporating the spectrum of technological innovations of today and the future, results in an adapted range of care offered by hospitals [56,58,61]. Thus, hospitals are increasingly becoming players on a health care platform on which various providers offer health care services depending on their competencies [53,65,73,77]. The schematic diagram illustrates the successive stages and draws 2 elementary trend lines along which the progress runs. Within this is the trend corridor. Along the time axis, both lines contribute to a higher value of care, with the contribution of technologies being lower than the value contribution of data. This is because technologies can provide additional benefits on their own. However, the higher overall benefit comes from the networked use of the data.

### Detected Trend Decelerators and Barriers

The analyses described numerous blockades and barriers that slow down the trends. Textbox 2 classifies these decelerators according to internal and external hurdles. External hurdles are defined as factors that are not under the hospitals’ control and usually have to be removed by a higher-level institution. This primarily refers to state institutions or self-governance. These aspects include the regulatory framework for health care services [60,65] or data protection regulations [40,46]; access to and the interoperability of data [41,46,49,55]; and an insufficient investment in hospital infrastructure [68,70,74], especially in technical equipment.

Internal hurdles are factors that occur within the hospital setting and can largely be solved there. The most frequently cited obstacle is a lack of knowledge of the use of digital applications [41,50,68,74]. The changes resulting from digital transformation are also accompanied by a shift in tasks and processes, some of which are not widely accepted by the workforce [44,63,67,75,76]. Furthermore, ensuring cybersecurity to avoid risks is an elementary component that clinics must accommodate to an increasing extent [20,42]. Finally, there is often a lack of perspective on how hospitals can position themselves in a new digital ecosystem of service providers as part of the digital transformation and how they can develop new business models and exploit the potential of digital provision [56,65,79].

**Type of hurdle and description of the results.**

**External hurdles**

- Regulation of service delivery [60,74]
- Data protection regulations [40,46]
- Uniformity of data structures [41,46,49,55,75]
- Lack of interfaces for data exchange [50,71,76]
- Insufficient investment in technical infrastructure [68,70,74]
- Lack of political understanding of appropriate frameworks [64,74]

**Internal hurdles**

- Inadequately qualified personnel [41,50,68]
- Low acceptance of substantially changing processes [44,63,67,75]
- Unwillingness to make large investments [38,55,63,68]
- Failure to ensure cybersecurity [20,42]
- Continuous training needs [49,55,59,71]
- Availability of suitable business models [56,65,79]

## Discussion

### Principal Findings

We included 44 studies in our analysis, drawing a picture of hospital trends. Subsequently, the trends in the German hospital care setting were discussed, validated, and refined through expert interviews. A wide range of aspects were considered, covering the use of new technologies, the implementation of process innovations, and behavior changes or role shifts among patients and hospital staff. The trends shown provide a framework for better assessing current and future developments from the perspective of clinics and other health care stakeholders. This allows for a snapshot of one’s own point of view or an assessment by higher-level authorities as to which changes in the framework conditions can accelerate or slow down the trends. For this purpose, trend barriers and developmental hurdles were also extracted from the studies. These can be used to derive policy options.

The trends described were not examined in terms of their positive or negative impact. Nevertheless, a positive trend is discernible in the development horizon, which is improving inpatient care and steering it in a patient-centered direction. Therefore, it is conceivable that not all trends will lead to a positive development for all stakeholders involved. The increased use of technology, automation, and AI systems will in some cases entail job losses or a restructuring of work [91]. Therefore, for hospital staff (employee representatives or trade unions), change through digital transformation is not necessarily associated with improvements. These aspects must also be considered. In contrast, digitization leads to the extensive aggregation of data, which can restrict people’s freedom of data self-determination. In addition, one-sided power positions of companies that have very large amounts of data at their disposal can lead to imbalances [92]. This can also preclude better patient care or unfair competition in the market [93]. The constant change in the course of the digital transformation of hospitals will in the future involve staff and decision makers in the qualification and further development of the range of services as the speed of change is increasing and the demands on service providers are continually rising [94]. The positive aspects of digital transformation should also be emphasized as they have the potential to prevent illnesses in the future, improve outcomes, and relieve staff of nonmedical tasks. In view of the impending shortage of skilled workers in health care professions around the hospital and beyond, this can unleash tied-up resources. Technical systems and algorithms can take over assisting tasks and, thus, optimize internal clinical processes as well as improve the entire spectrum of care from prevention to individualized therapy [95]. We are already seeing how technical innovations and digital applications are changing the professions in hospitals [96-98]. With the help of these tools, the high future demand for skilled workers can be partially compensated or substituted. In addition, dealing with innovations can lead to a higher attractiveness of the professions. The included studies tended to show a positive overall picture in which the opportunities dominate over the risks. The trend development was partly based on specific findings from Germany. However, overall, the trend perspectives can also be applied to other countries. In Europe, there are countries such as Switzerland, Austria, and the Netherlands, among others, that have similar health care system conditions. The analogs of the perspectives of digitally controlled hospital care can also be transferred to almost all other countries.

### Implications for Public Health and Future Medicine

Our study shows that there is enormous potential for public health to make care in hospitals more patient-centric as a result of digital transformation. This means that diseases can be detected and treated earlier. At the end of a successful transformation, there is a healthier population that takes advantage of the opportunities offered by preventative and precautionary health care. Clinics contribute to realizing this goal and must be prepared by decision makers for their changed role. As a result, comprehensive savings are possible, which can relieve the pressure on the health care system [99]. Strained health care systems such as those in Germany and other European countries could be relieved as a result. The shortage of specialists in inpatient care can be slowed down. For medical professions, this transformation means, on the one hand, relief through new technologies or support in diagnostics and therapy. Therefore, more time can be spent on advising and caring for patients, and the quality of care can be improved. In contrast, increasing digitization requires the learning of new skills and a change in working methods from physical to technical or technology-supporting activities. In the continuing education of physicians, it is essential that these competencies are sharpened. The increased interaction of patients, who, in the future, will collect their own data, monitor the progress of their illnesses with data support, and use mobile devices to contact hospitals, requires a high level of health literacy and digital competencies. School education should prepare children for this and also offer programs for older people to acquire the necessary knowledge. A low-threshold access to information and guidance should also be established.

### Limitations

The included studies already cover a comprehensive range of current and future trends. Nevertheless, the individual aspects may not have been considered. The analysis was primarily conducted from the perspective of inpatient service provision. The patient, as an important participant in the care process, was not explicitly examined. For example, sociological trends with regard to the use of digital applications could lead to findings that were not reflected [100]. These include the acceptance of new technologies and the willingness to use them. This aspect can also be extended to personnel in clinics. The distinction between trends, future forecasts, and expectations is not entirely clear-cut. Trends comprise medium-term developments in a relatively delimited subject area. We tried to apply this reference position as best as possible in our study. Some developments also refer to a longer time horizon as the initial situation varies greatly among countries. Thus, this study only showed the general developments that are possible in the next 5 to 10 years. The extent to which these developments can be realized or which other influencing factors accelerate or slow down individual aspects must be assessed individually for each health care system [49]. Therefore, a generalization to all health care systems is not conceivable. The German perspective could lead to minor distortions here in some cases. However, Germany’s reference role in many respects supports our argument. The reference period of the past 5 years covers a relatively short time horizon. We decided to carry out our study in this way as, in our view, studies conducted before 2016 with data from previous years are not informative about future trends [36,37]. The screening of databases also revealed that most studies on trends in the digitization of health care were published in the past 3 years (2018 to 2020) in particular. Owing to the COVID-19 pandemic, there may have been additional trends in 2020 that we did not explicitly include in our analysis [101]. As little, or at best rudimentary, research was available in the fall of 2020 when we conducted our database search, we excluded this option from our analysis. Nonetheless, we cannot rule out that new trends for clinics may have emerged as a result of the pandemic that have not yet been adequately considered in this work.

### Conclusions

In our study, we showed the influence of digital transformation on inpatient care. In the process, the high complexity of the interacting players became clear. We extracted 8 core trends from the international literature and validated them with experts in relation to the change in hospital care provision in Germany. We presented the interrelationships in a heuristic impact model. Our study highlights the barriers to successful transformation based on internal and external factors. We provide recommendations for action for decision makers on how public health can be improved by a digitally transformed hospital landscape and which hurdles need to be overcome.

## Appendix

Multimedia Appendix 1 Search terms.

Multimedia Appendix 2 Inclusion and exclusion criteria.

Multimedia Appendix 3 Data extraction form.

Multimedia Appendix 4 Literature overview.

Multimedia Appendix 5 Documentation of the scoping review.

## Abbreviations

AI: artificial intelligence
IoT: Internet of Things
PRISMA: Preferred Reporting Items for Systematic Reviews and Meta-Analyses
