# Supplementary material for: The Impact of Digital Transformation on Inpatient Care: Mixed Methods Study
Source: JMIR Public Health Surveill. 2023 Apr 21;9:e40622. doi: 10.2196/40622 (PMC10163407; doi:10.2196/40622)
Supplement: Multimedia Appendix 1 [file publichealth_v9i1e40622_app1.pdf]

Multimedia Appendix 1 Search terms

Database	Search terms 1	*AND*	Search terms 2	*AND*	Search terms 3
PubMed (Medline)	*"forecast" OR "foresight"*	+	*"hospital" OR "inpatient care"*	+	*"digitization" OR "digital transformation"*
EconLit	*"forecast" OR "foresight"*	+	*"hospital" OR "inpatient care"*	+	*"digitization" OR "digital transformation"*
EconBiz	*"forecast" OR "foresight"*	+	*"hospital" OR "inpatient care"*	+	*"digitization" OR "digital transformation"*
Science Direct	*"forecast" OR "foresight"*	+	*"hospital" OR "inpatient care"*	+	*"digitization" OR "digital transformation"*
