# Supplementary material for: The Impact of Digital Transformation on Inpatient Care: Mixed Methods Study
Source: JMIR Public Health Surveill. 2023 Apr 21;9:e40622. doi: 10.2196/40622 (PMC10163407; doi:10.2196/40622)
Supplement: Multimedia Appendix 2 [file publichealth_v9i1e40622_app2.pdf]

Multimedia Appendix 2 Documentation search strategy

search strategy

			inclusion criteria						exclusion criteria							
no	study	year	Trend related	Foresight related	Digital related	Technology related	Hospital related	Healthcare delivery related	COVID-19 related	Disease related	Discipline related	Treatment comparison related	Other healthcare sector related	Strong education related	Past related	Non-digital context
1	Abitol et.al.	2020	+	+	+	+	+	+	-	-	-	-	-	-	-	-
2	Aceto et.al.	2020	+	+	+	+	+	+	-	-	-	-	-	-	-	-
3	Adler-Milstein et.al.	2017	+	+	+	+	+	+	-	-	-	-	-	-	-	-
4	Aghdam et.al.	2021	+	+	+	+	+	+	-	-	-	-	-	-	-	-
5	Baslyman et.al.	2017	+	+	+	+	+	+	-	-	-	-	-	-	-	-
6	Bergey et.al.	2019	+	+	+	+	+	+	-	-	-	-	-	-	-	-
7	Berntsen et.al.	2019	+	+	+	+	+	+	-	-	-	-	-	-	-	-
8	Bhandari et.al.	2020	+	+	+	+	+	+	-	-	-	-	-	-	-	-
9	Blaser	2018	+	+	+	+	+	+	-	-	-	-	-	-	-	-
10	Blease et.al.	2020	+	+	+	+	+	+	-	-	-	-	-	-	-	-
11	Brink et.al.	2017	+	+	+	+	+	+	-	-	-	-	-	-	-	-
12	Bukowski et.al.	2020	+	+	+	+	+	+	-	-	-	-	-	-	-	-
13	Burkoski et.al.	2019	+	+	+	+	+	+	-	-	-	-	-	-	-	-
14	Cano et.al.	2017	+	+	+	+	+	+	-	-	-	-	-	-	-	-
15	Chen	2018	+	+	+	+	+	+	-	-	-	-	-	-	-	-
16	Chong et.al.	2020	+	+	+	+	+	+	-	-	-	-	-	-	-	-
17	Daneshmand et.al.	2017	+	+	+	+	+	+	-	-	-	-	-	-	-	-
18	Deiters et.al.	2018	+	+	+	+	+	+	-	-	-	-	-	-	-	-
19	Denicolai et.al.	2020	+	+	+	+	+	+	-	-	-	-	-	-	-	-
20	Desai et.al.	2019	+	+	+	+	+	+	-	-	-	-	-	-	-	-
21	Dreyer et.al.	2017	+	+	+	+	+	+	-	-	-	-	-	-	-	-
22	Eckert et.al.	2019	+	+	+	+	+	+	-	-	-	-	-	-	-	-
23	Eleftheriou et.al.	2018	+	+	+	+	+	+	-	-	-	-	-	-	-	-
24	Fang et.al.	2019	+	+	+	+	+	+	-	-	-	-	-	-	-	-
25	Farahani et.al.	2020	+	+	+	+	+	+	-	-	-	-	-	-	-	-
26	Fuller et.al.	2020	+	+	+	+	+	+	-	-	-	-	-	-	-	-
27	Galetsi et.al.	2019	+	+	+	+	+	+	-	-	-	-	-	-	-	-
28	Gryson	2018	+	+	+	+	+	+	-	-	-	-	-	-	-	-
29	Guntuku et.al.	2020	+	+	+	+	+	+	-	-	-	-	-	-	-	-
30	Kelly et.al.	2020	+	+	+	+	+	+	-	-	-	-	-	-	-	-
31	Ker et.al.	2018	+	+	+	+	+	+	-	-	-	-	-	-	-	-
32	Lee	2018	+	+	+	+	+	+	-	-	-	-	-	-	-	-
33	Mitterecker et.al.	2020	+	+	+	+	+	+	-	-	-	-	-	-	-	-
34	Neumann et.al.	2019	+	+	+	+	+	+	-	-	-	-	-	-	-	-
35	Palanica et.al.	2019	+	+	+	+	+	+	-	-	-	-	-	-	-	-
36	Perez et.al.	2016	+	+	+	+	+	+	-	-	-	-	-	-	-	-
37	Reda et.al.	2020	+	+	+	+	+	+	-	-	-	-	-	-	-	-
38	Ricciardi et.al.	2019	+	+	+	+	+	+	-	-	-	-	-	-	-	-
39	Serbanati	2020	+	+	+	+	+	+	-	-	-	-	-	-	-	-
40	Staib et.al.	2017	+	+	+	+	+	+	-	-	-	-	-	-	-	-
41	Tian et.al.	2019	+	+	+	+	+	+	-	-	-	-	-	-	-	-
42	Vallo et.al.	2019	+	+	+	+	+	+	-	-	-	-	-	-	-	-
43	Wang et.al.	2017	+	+	+	+	+	+	-	-	-	-	-	-	-	-
44	Zolbanin et.al.	2020	+	+	+	+	+	+	-	-	-	-	-	-	-	-
