# Supplementary material for: The Impact of Digital Transformation on Inpatient Care: Mixed Methods Study
Source: JMIR Public Health Surveill. 2023 Apr 21;9:e40622. doi: 10.2196/40622 (PMC10163407; doi:10.2196/40622)
Supplement: Multimedia Appendix 3 [file publichealth_v9i1e40622_app3.pdf]

Multimedia Appendix 3 Data extraction form

CONCEPT	DEFINITION
**AUTHOR**	Study first author
**YEAR**	The year the study was published
**COUNTRY**	The country where the study was published
**PUBLICATION TYPE**	The medium in which the study was published (conference proceedings, journal article, institutional publication)
**MAJOY TECHNOLOGY**	The authoritative innovation or technology described in the study.
**CORE OUTCOME**	The key findings of the Study.
**EXAMPLES OF APPLICATION**	The application examples shown in the study. Case studies and medical interventions were not included according to the exclusion criteria.
**INTRA-HOSPITAL APPLICATION CLASSIFICATION**	The classification of the areas of application within the hospital into three subgroups:<br>a. instruments/methods for capacity planning or control<br>b. Instruments/methods for process design<br>c. Instruments/methods for interaction between staff or patients
**SECTOR CLASSIFICATION**	The classification of linkage to other sectors of health care according to three subgroups:<br>a. within the hospital only<br>b. with another sector of health care<br>c. with multiple health care sectors

**TREND BARRIERS**	The hurdles or constraints identified in the study that potentially hinder the positive trend development.
