# Supplementary material for: The Impact of Digital Transformation on Inpatient Care: Mixed Methods Study
Source: JMIR Public Health Surveill. 2023 Apr 21;9:e40622. doi: 10.2196/40622 (PMC10163407; doi:10.2196/40622)
Supplement: Multimedia Appendix 4 [file publichealth_v9i1e40622_app4.pdf]

Multimedia Appendix 5 Literature overview

Study	Year	Major technology	Core outcome
Abitol et.al.	2020	Robotics use in the operating room	Potential for cost savings, shorter durations of stay, better clinical outcomes
Aceto et.al.	2020	Industry 4.0 technologies in the healthcare sector	Broad range of applications of IoT in medical care and the use of big data for better control
Adler-Milstein et.al.	2017	EHR deployment in the hospital	Use of broad EHR functionalities were only rolled out to a limited extent; potential to increase the depth of the application of EHR functions
Aghdam et.al.	2021	IoT deployment in the hospital	Potential for better interaction and communication with patients, more efficient use of data possible, training of users
Baslyman et.al.	2017	Data-driven process optimization	Activity-based process integration enables the better control of clinical processes; more efficient allocation of resources
Bergey et.al.	2019	Data-driven process optimization	Health information technology (HIS) supports nursing tasks and process design; better use of available resources
Berntsen et.al.	2019	Digital patient pathways	Using EHR data, patient pathways can be created and managed according to person-centered, integrated and proactive (PiP) care
Bhandari et.al.	2020	Robotics use in the operating room	AI supports the use of surgical robots and improves cooperation between humans and machines in the operating room
Blaser	2018	EHR deployment in the hospital	Efficiency of service providers increases through the systematic processing of patient data; clinic assumes the role of the (data) platform
Blease et.al.	2020	Application of machine learning	Improve digital health literacy, better communication, as well as promote technical understanding
Brink et.al.	2017	Data mining	Optimization of the use of resources in diagnostics, the use of big data and data mining for decision support and quality improvement
Bukowski et.al.	2020	Artificial intelligence in diagnostics	Potential for better diagnostics through AI, the need for appropriate interfaces to transfer data from other data sources, the expansion of infrastructure, additional investments
Burkoski et.al.	2019	Digitization of nursing care	Improvement in safety, time use, team collaboration, technical failures, patient engagement and acceptance
Cano et.al.	2017	Data-driven resource allocation	Networking of different data sources leads to better diagnostic results and the efficient planning and control of resources
Chen	2018	Data-driven resource allocation	Use of big data leads to better resource management via Net Relation Map (NRM) combined with importance-resistance analysis (IRA)
Chong et.al.	2020	Platform concepts	Decision support through AI and the advancement of personalized medicine through a children's health platform
Daneshmand et.al.	2017	Robotics use in the hospital	Versatile applications for robotics deliver numerous optimization and efficiency potentials

Deiters et.al.	2018	Digitization of the hospital	Digital control of services and secondary processes; qualification and change management for the implementation of digitalization
Denicolai et.al.	2020	Personalized medicine	Better predictive models shift the spectrum of services from intervention to prevention; new business models emerge for service providers
Desai et.al.	2019	Application of machine learning	Better planning and control of capacities and resources possible, through real-time data-driven decision-making
Dreyer et.al.	2017	Application of machine learning	AI and ML lead to better results in diagnostics, enable better quality, personalized medicine and the optimization of processes
Eckert et.al.	2019	IoT deployment in the hospital	AR offers added value in both treatment and training settings
Eleftheriou et.al.	2018	Data mining	Data-driven decision-making reduces risks and costs in complex (large) healthcare organizations
Fang et.al.	2019	Application of machine learning	AI and ML lead to better outcomes in diagnostics and therapeutics; personalized medicine improves the quality of care
Farahani et.al.	2020	IoT deployment in the hospital	IoT offers numerous opportunities for networking devices and sharing data, enabling better diagnostic and therapeutic decision-making
Fuller et.al.	2020	EHR deployment in the hospital	Data-driven interactions with patients can increase engagement and improve care; the expansion of the functionalities of EHR is needed
Galetsi et.al.	2019	Data mining	Algorithms are used to improve decision-making using big data; better care through personalized medicine
Gryson	2018	Platform concepts	Patient-centric care can lead to disruption and new business models with personalized digital apps
Guntuku et.al.	2020	Data mining	Early detection of the potential patient flow into clinics via social media; particularly relevant for special care settings, such as emergency departments
Kelly et.al.	2020	IoT deployment in the hospital	Both provider efficiency and patient outcomes are improved; more safety in care implementable
Ker et.al.	2018	Data-driven process optimization	Reduction in waiting times, costs and inconsistencies in the treatment process through the use of the HIS
Lee	2018	Digitization of the hospital	Use of digital technologies leads to higher patient engagement and better communication; user education is necessary
Mitterecker et.al.	2020	Application of machine learning	Better planning and control of resources (including blood transfusions) using ML; reliable forecasts of demand are possible
Neumann et.al.	2019	Digitization of the hospital	New providers and disruptive business models threaten established service providers; better financial results and competitive advantages through digitization
Palanica et.al.	2019	IoT deployment in the hospital	VR leads to better patient understanding, satisfaction, and engagement; the broad applicability of VR
Perez et.al.	2016	Data-driven process optimization	Processes, tasks and clinic capacities can be better controlled based on data; strong decision support
Reda et.al.	2020	Platform concepts	Using blockchain, all players in the supply chain in the healthcare market can interact in a better networked way; new business models are possible

Ricciardi et.al.	2019	Digitization of the hospital	State institutions can intervene in a steering manner to promote digital transformation; topics: funding, training, innovation support, quality enhancement
Serbanati	2020	EHR deployment in the hospital	Simulation of the patient's health status based on EHR data using an avatar; (decision-making) support for staff in providing care to the patient
Staib et.al.	2017	Data-driven process optimization	Increased survival rates and improved outcomes through optimized collaboration between emergency departments and wards using digital information distribution via dashboard
Tian et.al.	2019	Platform concepts	Technologies, such as AI, IoT and EHR improve care and control capabilities in connected digital health systems
Vallo et.al.	2019	Digitization of the hospital	Patients increasingly move from beneficiary to partner, greater involvement and new doctor–patient relationship
Wang et.al.	2017	Data mining	Better decision-making leads to better performance in the planning and execution of core processes; increased business value and competitive position
Zolbanin et.al.	2020	Data-driven resource allocation	Precise prediction of treatment courses and the length of stay is possible for the better planning and control of resources
