# Supplementary material for: The Impact of Digital Transformation on Inpatient Care: Mixed Methods Study
Source: JMIR Public Health Surveill. 2023 Apr 21;9:e40622. doi: 10.2196/40622 (PMC10163407; doi:10.2196/40622)
Supplement: Multimedia Appendix 5 [file publichealth_v9i1e40622_app5.pdf]

Multimedia Appendix 6 Documentation scoping review

study overview

no	study	year	T1	T2	T3	T4	T5	T6	T7	T8
1	Abitol et.al.	2020				x		x	x	
2	Aceto et.al.	2020		x	x	x		x		
3	Adler-Milstein et.al.	2017			x	x				
4	Aghdam et.al.	2021		x	x		x			
5	Baslyman et.al.	2017			x	x				
6	Bergey et.al.	2019				x	x			
7	Berntsen et.al.	2019		x	x	x	x		x	
8	Bhandari et.al.	2020				x		x	x	
9	Blaser	2018		x	x					x
10	Blease et.al.	2020	x				x			
11	Brink et.al.	2017			x			x	x	
12	Bukowski et.al.	2020		x	x	x		x		
13	Burkoski et.al.	2019	x			x			x	
14	Cano et.al.	2017		x	x				x	
15	Chen	2018			x	x				
16	Chong et.al.	2020							x	x
17	Daneshmand et.al.	2017				x		x		
18	Deiters et.al.	2018			x	x	x			
19	Denicolai et.al.	2020			x				x	x
20	Desai et.al.	2019			x	x		x		
21	Dreyer et.al.	2017				x			x	
22	Eckert et.al.	2019					x	x		
23	Eleftheriou et.al.	2018		x	x	x	x			
24	Fang et.al.	2019				x			x	
25	Farahani et.al.	2020					x	x	x	
26	Fuller et.al.	2020	x			x	x			
27	Galetsi et.al.	2019			x				x	
28	Gryson	2018					x		x	x
29	Guntuku et.al.	2020	x	x	x	x	x			x
30	Kelly et.al.	2020				x		x	x	
31	Ker et.al.	2018				x	x			
32	Lee	2018	x			x	x			
33	Mitterecker et.al.	2020			x	x				x
34	Neumann et.al.	2019					x	x		x
35	Palanica et.al.	2019	x				x	x		
36	Perez et.al.	2016			x	x	x			
37	Reda et.al.	2020						x		x
38	Ricciardi et.al.	2019					x	x	x	
39	Serbanati	2020			x	x		x	x	
40	Staib et.al.	2017			x	x			x	
41	Tian et.al.	2019		x	x		x	x		
42	Vallo et.al.	2019	x				x			
43	Wang et.al.	2017				x			x	x
44	Zolbanin et.al.	2020			x	x			x	
